# Clinicopathologic subtypes and compromise of lymph nodes in patients with breast cancer

**DOI:** 10.3332/ecancer.2014.448

**Published:** 2014-07-23

**Authors:** B Jaime Jans, M Nicolás Escudero, B Dahiana Pulgar, C Francisco Acevedo, R César Sánchez, A Mauricio Camus

**Affiliations:** 1 Department of Oncologic and Maxillofacial Surgery, Pontificia Universidad Católica de Chile, Marcoleta 352, Santiago 8330033, Chile; 2 Department of Haematology-Oncology, Pontificia Universidad Católica de Chile, Marcoleta 352, Santiago 8330033, Chile

**Keywords:** breast neoplasms, sentinel lymph node biopsy, lymph node dissection, triple-negative breast neoplasms

## Abstract

Breast cancer (BC) is currently a heterogeneous disease with variations in clinical behaviour. Classification according to subtypes has allowed progress in the individualisation of treatment. The objective of this study is to evaluate the risk of axillary node compromise in patients with BC, according to clinicopathologic subtypes. Materials and methods are a retrospective, descriptive-analytical study. All patients that had undergone surgery for invasive BC were included, with the study of sentinel lymph nodes (SLNs) at Hospital Clínico de la Pontificia Universidad Católica, between May 1999 and December 2012. The results showed 632 patients fulfilled the inclusion criteria, with the median age being 55 years (range: 28–95), and 559 (88.4%) patients presented with estrogen receptor and/or progesterone receptor positive tumours. Luminal A: 246 patients (38.9%), luminal B: 243 (38.4%), luminal not otherwise specified: 70 (11.1%) triple negative (TN): 60 (9.5%) and over expression of epidermal growth factor type 2 receptor (HER2 positive): 13 (2.1%). Luminal tumours displayed a greater risk of metastasis in the SLNs, but this difference was not statistically significant (*p* = 0.67). TN and HER2 positive tumours presented the greatest proportion of metastatic compromise in non-sentinel lymph nodes (non-SLNs) (57.1% and 50%, respectively). The presence of macrometastasis (MAM) in the SLN was associated with a greater risk of compromise of the non-SLN. Conclusions: Luminal tumours are the most frequent and present a greater proportion of axillary lymph node compromise, without being statistically significant. TN and HER2 positive tumours tend to have a higher axillary compromise; however, this was not statistically significant in either. Only the presence of MAM in SLNs displayed a statistically significantly association in the compromise of non-SLNs.

## Introduction

Breast cancer (BC) is the number one cause of cancer deaths in Chilean women. The estimated death rate in Chile is 14.5 per 100,000 women, followed by gallbladder and gastric cancer [1]. Early diagnosis and improved treatment have managed to improve the prognosis and increase the degree of healing from the disease. Currently, the majority of cases are diagnosed at localised stages of the disease and present an extended life span of ten years in greater than 80% [2]. In Chile, close to 95% of neoplasms are presented in localised stages (I, II, and III), and this percentage is also observed in our institution [3]. Therefore, management of the localised disease is the most frequent scenario.

The prognostic factors usually described are age, type and histologic grade, lymphovascular invasion, expression of hormonal and HER2 receptors, neoplasm size, and axillary compromise, the latter being the most important prognosis factor for BC [4].

Recently, the description of molecular subtypes of BC has demonstrated to play a role not only in prognosis but also in prediction. While systemic therapy is partially guided by the identification of these subtypes, it hasn’t been clearly demonstrated that this information affects the surgical management of the axilla. Due to possible morbidity of axillary lymph node dissection (ALND), many investigators who have evaluated, are limiting its recommendation. Recent studies promote a more conservative management of axillary disease, even in the presence of positive sentinel lymph nodes (SLNs) [5–6]. While trying to modulate surgical treatment, biological factors that help to determine the risk of axillary disease have been investigated with the goal of avoiding ALND [7–8].

The objective of this study was to evaluate axillary compromise in patients with BC, according to their clinicopathologic profile. The specific objectives were: the classification of tumours according to subtypes, the correlation of these subtypes with compromise of SLNs and of non-sentinel lymph nodes (non-SLNs) post ALND, and the evaluation of risk factors of compromise in non-SLNs post ALND.

## Patients and methods

We carried out a retrospective, descriptive-analytical study. All patients who underwent surgery for invasive BC between May 1999 and December 2012 at Hospital Clínico de la Pontificia Universidad Católica de Chile were included, with biopsy of SLN. Patients excluded were those with microinvasive carcinoma (T1mic), those treated with neoadjuvant therapy, and those with tumours for which there was not enough information for classification in luminal or non-luminal clinicopathologic subtypes. The technique for the biopsy of the SLN used in the majority of patients was a mixed technique (lymphoscintigraphy with injection of Nanoscint® marked with Tecnecio99m, with the use of a radioactivity mapping intraoperative probe and 1% patent blue dye). ALND was performed on patients with SLN metastasis, considering variables such as age, comorbidities, size of metastasis, use of neoadjuvant therapies, and with consensual decision of the oncologic board [9], information was obtained from a prospective registry of medical records and biopsies from the Department of Pathologic Anatomy of our centre.

In addition to demographic variability, we obtained anatomopathologic information: histologic types, and histologic grade (HG) differentiation according to classification by Elston and Bloom, modified by Elston and Ellis [10], study of ER and PR, on a scale of 0–100%, HER2 on a scale of + to +++, all through methods of immunohistochemistry (IHC). Considered estrogen receptor (ER) or progesterone receptor (PR) positive were those with staining ≥1%. With regard to HER2: + negative, ++ uncertain, additionally evaluated by immunofluorescence *in situ* (FISH), and +++ positive.

The clinicopathologic subtype of the tumour was defined by the following elements: Luminal A (ER positive and PR ≥20%, HG 1–2, HER2 negative); Luminal B (ER positive and PR <20%, HG 3 and/or HER2 positive), triple negative (TN) (ER, PR, and HER2 negative), HER2+ (ER and PR negative, HER2 positive) [11]. During the length of the study Ki67 was not available, therefore HG was used as a parameter associated to the value of Ki67 [12]. Those greater than 2 mm were considered macrometastasis (MAM), micrometastasis (MM) were between 0.2 and 2 mm, and submicrometastasis (SMM) were those up to 0.2 mm [13].

For the statistical analysis, the program SPSS 20.0 was used. Descriptive statistics were used. To compare tests, parametric and nonparametric hypothesis tests were done according to the determination of normality. A level of confidence of 95% with *p* < 0.05 was considered statistically significant.

## Results

During the period of analysis, 773 patients had SLN biopsies, and 632 fulfilled the inclusion criteria. Their characteristics are shown in Table 1. The median age of the patients was 55 years (range: 28–95), 518 (82%) of tumours corresponded to ductal adenocarcinomas, 409 (64.7%) were less than 2 cm, and 559 (88.4%) presented positive ER and/or PR.

The most frequent clinicopathologic subtypes were luminal A with 246 (38.9%), followed by luminal B with 243 (38.4%). There were 70 luminal tumours (11.1%) whose subtype was unable to be specified. Among the non-luminal tumours, TN corresponded to 60 (9.5%), and overexpression of HER2 to only 13 (2.1%) of the patients.

The median of detected SLNs was two (range: 1–11). Metastasis in SLNs were detected in 207/632 cases (32.8%), of which 140 (67.6%) corresponded to MAM, 35 (16.9%) to MM, and 32 (15.5%) to SMM. Tumours with a luminal A profile presented the highest proportion of metastatic compromise in the SLN (34.6%) and TN the lowest (25%), although there were no statistically significant differences found among distinct profiles with regard to SLN compromise (*p* = 0.67) (Table 2).

Of the 207 cases with metastasis in the SLN, 184 (88.9%) were treated with ALND. Additional metastasis was found in non-SLNs in 79 (42.9%) of patients. The frequency of metastasis in non-SLNs was 47.1% in patients with MAM (66/140), 28% in MM (7/25), and 31.6% in SMM (6/19). TN and HER2 positive tumours presented the highest proportion of metastatic compromise in non-SLNs (57.1% and 50%, respectively). However, no statistically significant differences were found in metastatic compromise of non-SLNs according to the clinicopathologic profile (Table 3). When subdividing luminal B into HER2 positive and HER2 negative, the first presented significantly lower non-SLN compromise (25% versus 53.8%; *p* = 0.04).

To determine the frequency of modification in the lymph node stage post ALND, patients were grouped according to clinicopathologic profiles into luminals and non-luminals. From the 166 luminal tumours dissected, 27 changed their lymph node stage to pN2 and six to pN3. This resulted in a percentage of modification of 19.9%, and of the 18 non-luminal tumours dissected, three changed their lymph node stage to pN2 and two to pN3. This resulted in a percentage of modifications of 27.8%. This difference was not statistically significant (*p* = 0.43).

When subdividing patients according to the size of metastasis, in patients with MAM, the modification of the lymph node stage was 24.2% versus 33.3% in luminals and non luminals, respectively (*p* = 0.44). There was no significant difference when correlating results with the size of tumour (*p* = 0.15) (Table 4). In patients with MM and SMM, the modification of the lymph node stage was 18.6% versus 66.7% in luminals and non-luminals, respectively, without statistically significant differences (Table 5).

Finally, we evaluated whether age, tumour size, the size of metastasis in the SLN, or the subtype correlated with the presence of metastasis in non-SLNs. Only the presence of MAM in SLNs displayed a statistically significant association in the compromise of non-SLNs in ALND (*p* = 0.04) (Table 6).

## Discussion

The study of SLNs is currently the technical standard used to determine the compromise of axillary lymph nodes in patients with BC and clinically negative axilla [14–17], with much lower morbidity than ALND [18–21]. This has allowed certain patients to use ALND as a therapeutic procedure and not a diagnostic one. The study of SLNs allows for pathological staging of the axilla (pN), identifying patients that do not have axillary node compromise, and hence do not require additional surgeries; and patients with nodal metastasis who could benefit from an ALND afterward. However, the real benefit of ALND is not all that clear [6, 22–24] and has been questioned in patients with positive SLNs with no effect of non-SLNs, a situation present in 50–70% of the cases [25]. Various clinical studies have established the safety of omitting ALND in patients with up to two metastatic SLNs, treated with conservative surgery (partial mastectomy and radiotherapy), and systemic therapy (chemotherapy and/or hormone therapy) [4].

The majority of nomograms that have been developed and validated to predict the risk of compromise of the SLN and non-SLN are mainly based on classic histopathologic parameters [26–28]. The integration of information contributed to the knowledge of the molecular profile of the tumour could help improve the prediction of these models. In 2000, Perou *et al* [29] described the gene expression pattern of invasive BC, thus making it possible to establish molecular classification of BC, currently represented mainly by four subtypes: luminals A and B, HER2, and TN, each with defined characteristic and prognosis. The immunohistochemical characterisation allowed an approximation of these molecular subtypes to be reached, establishing four subtypes based on expression of ER, PR, HER2, and the presence of cell proliferation indicators, such as Ki67 and/or HG. Defining molecular or clinicopathologic subtypes has become a fundamental parameter when deciding adjuvant therapies, evaluating prognosis, and eventually defining the surgical proximity to the axilla, based on the prediction of lymph node disease.

In this study, we have sought to evaluate axillary compromise in patients with BC, according to their clinicopathologic profile defined by the study of IHC and HG. As in other series, the majority of our patients presented luminal neoplasms (88.4%: A 38.9% and B 38.4%), 9.5% were TN tumours, and only 2.1% HER2-enriched. In 11.1%, the state of HER2 was unknown. This is because before the year 2005 this study was not regularly carried out at our centre.

For the classification of clinicopathologic subtypes, we used a modification of the definitions proposed by the last consensus at St. Gallen [24], replacing the measuring of Ki67 with HG, to differentiate luminal A (HG 1–2) and luminal B (HG3). This was because we did not carry out Ki67 analysis for all of our patients, since this was not part of our daily work protocol. Many projects have demonstrated that HG was a good parameter of cellular proliferation, accurately correlating with the values of Ki67 [10–12].

We found that SLN compromise was higher in tumours of luminal A (34.6%) and lower in TN (25%), without reaching a statistically significant difference (*p* = 0.16). This coincides with findings from other authors. For example, Gann *et al* [30] analysed prediction factors for axillary nymph node compromise. The absence of ER and PR was associated with a lower risk of nodal compromise (OR = 0.82). Lu *et al* [31] determined a model for axillary lymph node prediction including tumour size, lymphovascular invasion (LVI), and biological subtype (based on ER. HER2, and HG). When comparing tumours of equal size and LVI state, patients with positive ER and negative HER2 had the highest probability of metastatic lymph node compromise, while ER and HER2 negative had the lowest risk. Bevilacqua *et al* [32], from Memorial Sloan Kettering Cancer Centre elaborated a normogram using nine clinical and pathological variables in 3,786 biopsies of SNL, and found that the rate of metastasis in SLNs was higher in luminal tumours. Viale *et al* [33] studied prediction factors for compromise of SNLs in 4,352 patients. They found that this was directly associated with tumour size, multifocality, and the presence of peritumoural vascular invasion. In addition, there was an inverse association between favourable histology and the absence of PR. On the other hand, Crabb *et al* [34] found that basal subtype had the lowest risk of lymph node compromise (OR = 0.53).

With regard to non-SLN compromise, we found that TN and HER2 positive tumours presented the highest proportion of metastatic compromise (57.1% and 50%, respectively). However, no statistically significant differences were found according to subtypes (Table 3); this might be due to the sample size of our series. Many authors have evaluated prediction factors of compromise in the non-SLNs, without finding statistical significance in multivariate analysis for biological markers (ER, PR, HER2) [35]. In a study by Degnim *et al* [36], meta-analysis was done to evaluate clinicopathologic factors of metastasis in non-SLNs. Eleven studies met the inclusion criteria. It was found that the presence of MAM, extranodal invasion, tumour of size >2 cm, two or more positive SLNs, and the presence of LVI in the primary tumour, were associated with more than double the risk. Finally, Kwon *et al* [35] analysed clinicopathologic and biological factors predicting compromise of non-SLNs in patients with SLN metastasis. They found that MAM, the presence of two or more positive SLNs, and extranodal extension were independent predictors of non-SLN metastasis. None of the classic biological markers, including hormone receptors HER2 and Ki67, were useful predictors. In addition, they found that patients with the three characteristics like the micrometastasis, only one positive SLN, and the absence of extranodal extension, had the possibility of additional axillary lymph node metastasis, which is only 5.6%. In our analysis, only the presence of MAM in the SLN was significantly associated with more non-SLN, which is in accordance with what has been previously described.

## Conclusion

In our study, luminal tumours were the most frequent and presented a greater proportion of axillary lymph node compromise, without being statistically significant. Even though there was a trend to a more extensive axillary compromise in TN and HER2 positive tumours, this was not statistically significant in either. Only the presence of MAM in SLNs displayed statistically significant association with the compromise of non-SLNs in ALND. It seems important to take into account the clinicopathologic profile when designing clinical studies that evaluate the management of the axilla, since prospective studies that support non-ALND pose that in its majority, the luminal tumours, the TN and HER2 enriched tumours are underrepresented. Our study shows a trend of greater axillary node compromise in non-luminal tumours, when the SLN is affected, as was reported in other series. However, our results should be evaluated with caution, due to the low number of patients with tumours of this subtype. Better conclusions should be drawn from prospective studies and their clinical results.

## Conflicts of interest

The authors have no conflicts of interest.

## Figures and Tables

**Table 1.** Clinical and pathological characteristics of 632 patients with breast cancer in sentinel lymph node study.

Variable	*n*	%
**Age (years)**		
Median (range)	55 (28–95)	-----
**Histology**		
Ductal	518	82.0
Lobular	47	7.4
Other	67	10.6
**T State**		
1	409	64.7
2	204	32.3
3	16	2.5
4	3	0.5
**ER and/or PR**		
Positive	559	88.4
Negative	73	11.6
**HER2**		
Positive	56	8.9
Negative	506	80.1
Unknown	70	11.1
**HG 3**		
Yes	211	33.4
No	377	59.7
Unknown	44	7.0

ER: estrogen receptor,

PR: progesterone receptor,

HG: histologic grade.

**Table 2.** Sentinel lymph node (SLN) compromise according to clinicopathologic subtype and size of metastasis.

			SLN+					
Clinicopathologic subtype	*n*	%	MAM	MM	SMM	Total	%	p*
Luminal A	246	38.9	54	17	14	85	34.6	
Luminal B	243	38.4	56	12	12	80	32.9	0.69
Luminal not specified	70	11.1	15	4	4	23	32.9	0.79
HER2	13	2.1	3	0	1	4	30.8	0.78
Triple negative	60	9.5	12	2	1	15	25.0	0.16
**Total**	632	100.0	140	35	32	207	32.8	

MAM: macrometastasis,

MM: micrometastasis,

SMM: submicrometastasis.

* *p* value calculated based on chi-square test.

All profiles were compared to luminal A.

**Table 3.** Patients with positive SLN, non-SLN compromise according to clinicopathological subtype.

Metastasis in SLN		Patients with ALND		Patients with nonSLN+		
Molecular Profile	*n*	*n*	%	*n*	%	p*
Luminal A	85	75	88.2	31	41.3	----
Luminal B	80	71	88.8	33	46.5	0.53
Luminal not specified	23	20	87.0	5	25.0	0.18
HER2	4	4	100.0	2	50.0	0.73
Triple negative	15	14	93.3	8	57.1	0.27
Total	207	184	88.9	79	42.9	

SLN: sentinel lymph node,

non-SLN: non sentinel lymph node,

ALND: axillary lymph node dissection.

* *p* value calculated based on chi-square test.

All profiles were compared to luminal A.

**Table 4.** Metastatic compromise of non-SLN, percent of modification to phase pN2 and pN3, in patients with MAM,according to clinicopathologic subtype and tumour size (*n* = 140 patients).

			Phase N post ALND				
Phase N pre ALND	With ALND	nonSLN +	pN1a	pN2	pN3	% modification	p*
**LUMINAL**	125	56	26	**24**	**6**	24.2	…
Tu ≤ 2 cm	53	27	17	**8**	**2**	17.0	
Tu > 2 cm	72	30	9	**18**	**4**	29.2	0.15
**NO LUMINAL**	15	8	3	**3**	**2**	33.3	0.44
Tu ≤ 2 cm	9	5	3	**1**	**1**	22.2	
Tu > 2 cm	6	3	0	**2**	**1**	50.0	0.26

non-SLN: non sentinel lymph node,

MAM: macrometastasis,

ALND: axillary lymph node dissection.

* *p* value calculated based on chi-square test.

**Table 5.** Metastatic compromise in non-SLN and percent of modifications to phase pN2 and pN3, in patients with MM and SMM, according to subtypes and tumour size (*n* = 44 patients).

			Phase N post ALND				
Phase N pre ALND	With ALND	nonSLN+	pN1a	pN2	pN3	% modification	p*
**LUMINAL**	41	12	6	1	0	18.6	
Tu ≤ 2 cm	25	4	1	1	0	8.0	
Tu > 2 cm	16	7	5	0	0	31.3	0.05^
**NO LUMINAL**	3	2	2	0	0	66.7	0.05^
No sorting by size made							

NonSLN: non-sentinel lymph node,

MM: micrometastasis,

SMM: submicrometastasis,

ALND: axillary lymph node dissection.

* p value calculated based on chi-square test.

^ Invalid significance calculated by chi-square test, due to low number of cases.

**Table 6.** Risk factors associated with the presence of metastasis in non-SLN in patients with ALND (*n* = 184).

	nonSLN + (*n* = 79)	nonSLN − (*n* = 105)	*p*
**Age (years)**			
Median (range)	55 (32–84)	53 (29–79)	0.45
**T**			
<2 cm	36	51	0.68
>2 cm	43	54	
**SLN +**			
MAM	66	74	**0.039**
MM and/or SMM	13	31	
**Molecular Profile**			
Luminal	69	97	0.25
No Luminal	10	8	
